# Pudendal Nerve Pulsed Radiofrequency Effectively Alleviates Perineal Pain in Interstitial Cystitis/Bladder Pain Syndrome: A Retrospective Study

**DOI:** 10.1155/prm/9320012

**Published:** 2025-12-06

**Authors:** Yiming Liu, Ou Wu, Yifan Yang, Shunan Xie, Yi Feng

**Affiliations:** ^1^ Department of Pain Medicine, Peking University People’s Hospital, Beijing, China, pku.edu.cn

**Keywords:** interstitial cystitis/bladder pain syndrome, perineal pain, pudendal nerve, pulsed radiofrequency

## Abstract

**Objectives:**

Interstitial cystitis/bladder pain syndrome (IC/BPS) is a refractory chronic pelvic pain disorder. In patients presenting with perineal pain, quality of life is severely compromised due to the lack of effective analgesic interventions. This study aimed to evaluate the efficacy and safety of pudendal nerve pulsed radiofrequency (PRF) for managing perineal pain in IC/BPS patients.

**Methods:**

We conducted a retrospective analysis of 51 female IC/BPS patients who underwent pudendal nerve PRF treatment at Peking University People’s Hospital between January 2020 and May 2024. Therapeutic outcomes were evaluated using validated metrics including the visual analog scale (VAS), interstitial cystitis problem index (ICPI) and symptom index (ICSI), hospital anxiety and depression scale (anxiety: HADS‐A and depression: HADS‐D), and pain catastrophizing scale (PCS) to comprehensively assess treatment efficacy on perineal pain and associated psychosocial comorbidities.

**Results:**

The VAS score decreased from 8.0 (7.0, 9.0) preoperatively to 4.0 (2.0, 6.0) at 6 months postoperatively (*p* < 0.0001). Both ICPI and ICSI scores decreased from 14.0 (12.0, 15.5) and 15.0 (12.0, 18.0) preoperatively to 8.0 (5.5, 11.0) and 8.0 (6.0, 13.0) at 6 months postoperatively, respectively (*p* < 0.0001). HADS‐A and HADS‐D scores decreased from 8.0 (4.5, 12) and 7.0 (4.5, 11.5) preoperatively to 5 (2.5, 9) and 3.0 (1.5, 7.0) at 6 months postoperatively, respectively (*p* < 0.05). The PCS score decreased from 36.58 ± 10.93 preoperatively to 13.46 ± 6.80 at 6 months postoperatively (*p* < 0.0001). The outcomes of pudendal nerve diagnostic blocks showed positive correlations with postoperative improvements in VAS, ICPI, and ICSI at 6 months. A small number of patients experienced transient buttock puncture site pain and acute urinary retention postoperatively, all of which resolved spontaneously without serious adverse reactions.

**Conclusions:**

Pudendal nerve PRF demonstrates significant therapeutic efficacy in managing perineal pain among patients with IC/BPS. The outcomes of pudendal nerve diagnostic blocks exhibit predictive value for the effectiveness of pudendal nerve PRF in alleviating IC/BPS‐associated perineal pain.

## 1. Introduction

Interstitial cystitis/bladder pain syndrome (IC/BPS) is a clinical syndrome characterized by intermittent pelvic and/or perineal pain accompanied by urinary frequency, urgency, and pain during bladder filling. Pathological hallmarks include idiopathic chronic bladder wall inflammation and interstitial fibrosis. Epidemiological studies report a female prevalence of 0.83%–2.71%, approximately 5–10 times higher than males [1]. The pathogenesis of IC/BPS remains unclear but may involve multifactorial etiologies such as occult infections, bladder mucosal barrier alterations, mast cell activation, autoimmune responses, and peripheral/central neural sensitization [2]. Perineal pain in IC/BPS typically manifests as burning, lancinating, or stabbing sensations, demonstrating classic neuropathic features. Increased density of bladder sensory nerve fibers in IC/BPS patients suggests that neural sensitization may mediate pain pathophysiology [3].

IC/BPS lacks disease‐modifying therapies. Current management primarily relies on intravesical drug instillation, hydrodistention, and oral medications (M‐receptor antagonists and anti‐inflammatory analgesics) for symptomatic relief. However, these interventions fail to address neural sensitization, resulting in inconsistent analgesic efficacy. Effective pain control remains a critical unmet need for IC/BPS patients, underscoring the imperative to develop novel therapeutic strategies. Neuromodulation techniques targeting neural sensitization pathways may offer potential for managing IC/BPS‐associated perineal pain. Gonzalez et al. demonstrated that pudendal nerve stimulation increased bladder capacity and compliance in cyclophosphamide‐induced IC/BPS rat models [4]. Peters et al. further reported significant symptomatic improvement, including perineal pain reduction, following pudendal neuromodulation in IC/BPS patients [5]. These findings collectively suggest that targeted pudendal nerve modulation represents an emerging therapeutic strategy for IC/BPS‐associated perineal pain.

Pulsed radiofrequency (PRF), a well‐established neuromodulation technique, delivers pulsed electrical currents via electrodes to neural tissues, thereby reducing neuronal excitability and producing significant analgesic effects. Widely employed in treating various neuropathic pain conditions, PRF is clinically recognized for its safety, minimal invasiveness, efficacy, and cost‐effectiveness [6]. In 2016, Ozkan et al. reported substantial perineal pain reduction in two IC/BPS cases following pudendal nerve PRF [7]. Building on these findings, our team conducted a pilot clinical study applying pudendal nerve PRF to IC/BPS patients, observing effective perineal pain relief in the majority of cases without procedure‐related complications. Through retrospective analysis of these clinical data, this study aims to demonstrate the therapeutic efficacy and safety profile of pudendal nerve PRF for IC/BPS‐associated perineal pain, proposing a novel neuromodulatory approach for this challenging condition.

## 2. Methods

### 2.1. Ethical Approval

This study received ethical approval from the Ethics Committee of Peking University People’s Hospital (Approval no. 2025PHB042‐001).

### 2.2. Patients

We conducted a retrospective analysis of 51 female patients with IC/BPS‐associated perineal pain treated at the Department of Pain Medicine, Peking University People’s Hospital between January 2020 and May 2024.

### 2.3. Inclusion Criteria

1. Aged 18–80 years.
2. Diagnosis of IC/BPS meeting European Society for the Study of Interstitial Cystitis (ESSIC) criteria [8].
3. Presence of perineal pain (visual analog scale [VAS] ≥ 3) with ≥ 50% pain relief following pudendal nerve diagnostic block.
4. Failure of behavioral therapy, and inadequate response to at least two guideline‐recommended therapies as per American Urological Association (AUA) guidelines, including oral medications (monotherapy or combined with intravesical agents) targeting glycosaminoglycan layer replenishment, anti‐inflammatory effects, analgesia, or muscle relaxation [9];
5. Completion of pudendal nerve PRF.
6. Full baseline assessment and 6‐month postoperative follow‐up.
7. Written informed consent was obtained.

### 2.4. Exclusion Criteria

1. History of pelvic surgery.
2. Presence of pelvic malignancies.
3. Receipt of additional therapies during follow‐up (e.g., sacral nerve stimulation, oral Chinese herbal medicine, and acupuncture).

### 2.5. Surgical Procedure

All procedures were performed by a single senior pain physician with extensive expertise in chronic pelvic pain management.

### 2.6. Pudendal Nerve Diagnostic Block

**Figure 1:**
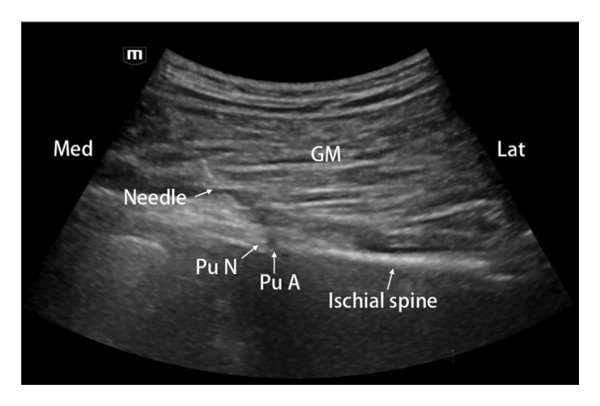
The puncture pathway diagram for the pudendal nerve diagnostic block. GM: gluteus maximus; Pu A: pudendal artery; Pu N: pudendal nerve.

Patients were positioned prone with sterile draping. A low‐frequency (2–5 MHz) convex ultrasound transducer (Mindray, China) was used to delineate the sciatic notch boundary, visualizing the sacrospinous ligament, sacrotuberous ligament, and pudendal artery at the ischial spine level. Following local anesthesia, a 22G needle (Tuoren, China) was advanced in‐plane through the sacrotuberous ligament to a position medial to the pudendal artery (Figure 1). After confirming negative aspiration, 5 mL of 1% lidocaine was injected. Patients’ self‐assessed pain relief percentage (0%: no relief; 100%: complete relief) 10 min postinjection. Bilateral pudendal nerve diagnostic blocks were performed for patients with bilateral perineal pain. Procedural success required both sensory anesthesia within the pudendal nerve distribution and ≥ 50% pain reduction, establishing eligibility for subsequent PRF therapy.

### 2.7. Pudendal Nerve PRF

Patients were positioned prone with sterile draping. A 22G, 10‐cm needle (5‐mm active tip; Beiqi, China) was advanced following the ultrasound‐guided pudendal nerve block protocol. Upon confirming needle tip placement medial to the pudendal artery (Figure 1), the electrode was connected to a radiofrequency generator (Beiqi, China). Sensory testing (50 Hz) was performed to refine needle positioning; paresthesia reproduction in the painful perineal region at ≤ 0.3 V confirmed proximity to the pudendal nerve. Motor testing (2 Hz) ensured the absence of ipsilateral limb contraction at ≥ 2 V, excluding sciatic nerve involvement. PRF was then initiated with parameters: 42°C, 2 Hz, 20‐ms pulse width, and 900‐s duration. Bilateral pudendal nerve PRF was administered for symmetrical pain presentations.

### 2.8. Data Collection

Study data were extracted from the electronic medical record (EMR) system and the chronic pelvic pain follow‐up registry of Peking University People’s Hospital. All follow‐up assessments were uniformly collected by a single physician assistant through electronic questionnaires administered to patients, spanning preoperative and 1‐, 3‐, and 6‐month postoperative intervals.

The primary outcome was assessed using the VAS (0 = no pain; 10 = worst imaginable pain). Secondary outcomes included the following: O’Leary–Sant Scores, comprising the interstitial cystitis problem index (ICPI) and symptom index (ICSI) to evaluate IC/BPS severity, hospital anxiety and depression scale (HADS) with subscales for anxiety (HADS‐A) and depression (HADS‐D), pain catastrophizing scale (PCS), pain sensitivity questionnaire (PSQ), and procedure‐related complications (local bleeding, infection, nerve injury, urinary/defecatory dysfunction, and other serious adverse events).

### 2.9. Statistical Analysis

Data analysis was performed using SPSS 27.0 (IBM Corp., USA). Continuous variables with normal distribution are expressed as mean ± standard deviation ($\overset{¯}{X}$ ± SD), while non‐normally distributed data are presented as median (interquartile range [IQR]). Categorical variables are reported as percentages. Longitudinal changes in VAS, ICPI, ICSI, HADS, and PCS scores before and after pudendal nerve PRF were analyzed using repeated‐measures ANOVA (for normally distributed data) or the Friedman test (for nonparametric data), with Bonferroni‐adjusted pairwise comparisons for post hoc analysis.

Delta values were calculated as

(1) ΔVAS=VAS pre−op−VAS post−op 6 month,ΔICPI=ICPI pre−op−ICPI post−op 6 month,ΔICSI=ICSI pre−op−ICSI post−op 6 month.

Correlation analyses between ΔVAS/ΔICPI/ΔICSI and diagnostic block outcomes, HADS, PCS, and PSQ scores, were conducted using Pearson’s test (normally distributed variables) or Spearman’s rank correlation (nonparametric variables). A two‐tailed *p* < 0.05 was considered statistically significant.

## 3. Results

### 3.1. Patients

**Figure 2:**
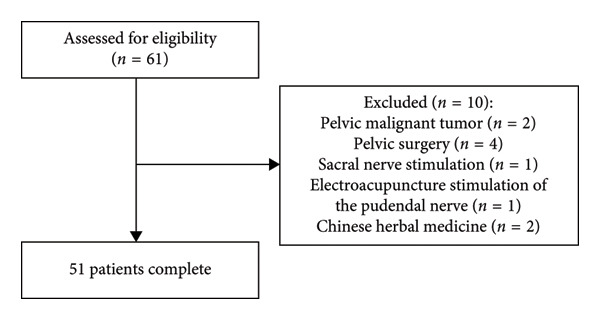
Flowchart of patient selection.

A total of 61 female patients with refractory IC/BPS‐associated perineal pain were initially enrolled. Among them, 6 patients were excluded due to pelvic malignancies or prior pelvic surgery. During follow‐up, 4 additional patients were excluded for receiving nonprotocol therapies: 1 underwent sacral nerve stimulation, 1 received pudendal nerve electroacupuncture, and 2 utilized oral Chinese herbal medicine (Figure 2). Consequently, 51 patients were included in the final analysis, with a mean age of 59.32 ± 6.64 years and pain duration of 4.51 ± 2.57 years (Table 1).

**Table 1:** Patients’ baseline characteristics.

Variables	Value	
Numbers	51	

Gender	Female	

Age (years)	59.32 ± 6.64	

Body mass index (kg/m^2^)	24.12 ± 3.67	

Duration of pain *n* (%)	< 1 (years)	11 (21.6%)
	1–3 (years)	24 (47.0%)
	> 3 (years)	16 (31.4%)

Pain site *n* (%)	Dorsal nerve of the clitoris	8 (15.7%)
	Perineal nerve	31 (60.8%)
	Inferior rectal nerve	5 (9.8%)
	Suprapubic region	26 (51.0%)
	Lumbosacral	10 (19.6%)
	Lower limbs	5 (9.8%)

Unilateral/bilateral *n* (%)	Unilateral	42 (82.4%)
	Bilateral	9 (17.6%)

Pain characteristics *n* (%)	Burning	33 (64.7%)
	Stabbing	22 (43.1%)
	Throbbing	18 (35.3%)
	Splitting	16 (31.4%)
	Aching	10 (19.6%)

Phenotypes *n* (%)	Hunner’s lesion type	8 (15.7%)
	Non‐Hunner’s lesion type	43 (84.3%)

Diagnostic block of the pudendal nerve *n* (%)	50% ≤ degree of mitigation < 70%	5 (9.8%)
	70% ≤ degree of mitigation < 90%	27 (52.9%)
	Degree of mitigation ≥ 90%	19 (37.3%)

*Note:* Values are presented as numbers (%) for categorical variables and mean ± SD for continuous variables.

#### 3.1.1. Pain Scores

**Figure 3:**
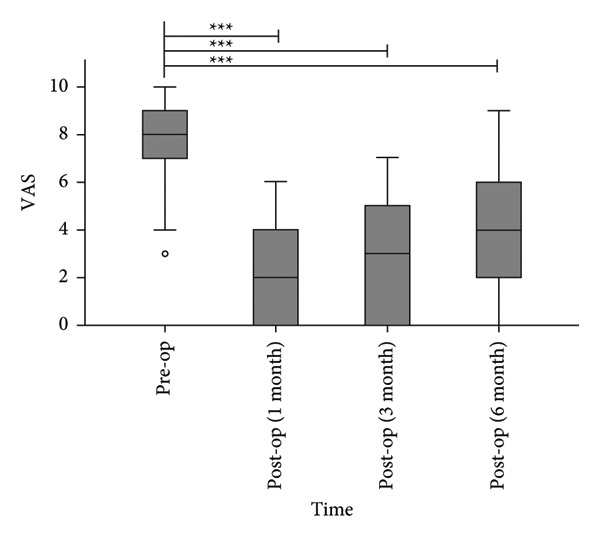
Postoperative VAS scores were significantly lower than preoperative levels. ^∗∗∗^ *p* < 0.0001, compared to pre‐op.

The median VAS scores decreased from 8.0 (7.0, 9.0) preoperatively to 2.0 (0.0, 5.5), 3.0 (0.0, 4.0), and 4.0 (2.0, 6.0) at 1, 3, and 6 months postoperatively (Figure 3). All postoperative VAS scores demonstrated statistically significant reductions compared to baseline (*p* < 0.0001).

#### 3.1.2. O’Leary–Sant Scores

The median ICPI scores decreased from 14.0 (12.0, 15.5) preoperatively to 4.0 (2.0, 7.5), 5.0 (3.0, 9.5), and 8.0 (5.5, 11.0) at 1, 3, and 6 months postoperatively (Figure 4(a)), with all postoperative timepoints demonstrating statistically significant reductions compared to baseline (*p* < 0.0001). Similarly, the median ICSI scores decreased from 15.0 (12.0, 18.0) preoperatively to 6.0 (3.0, 8.5), 6.0 (3.0–10.0), and 8.0 (6.0–13.0) at 1, 3, and 6 months postoperatively (Figure 4(b)), showing significant improvements at all follow‐up intervals (*p* < 0.0001).

Figure 4Postoperative ICPI scores (a) and ICSI scores (b) were significantly reduced compared to preoperative levels. ^∗∗∗^ *p* < 0.0001, compared to pre‐op.

**Figure:**
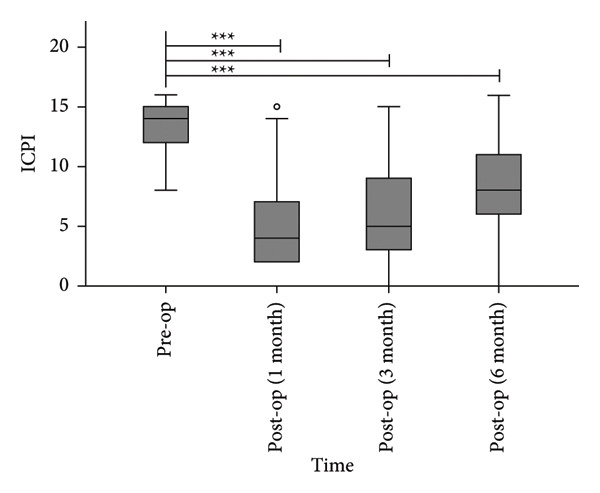
(a)

**Figure:**
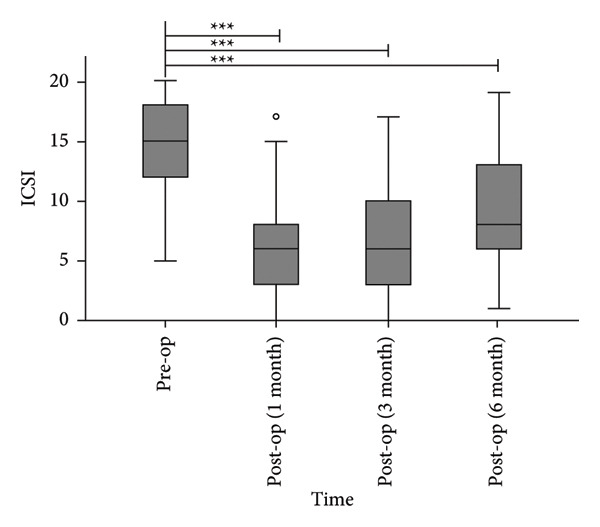
(b)

#### 3.1.3. Anxiety and Depression

The median HADS‐A scores decreased from 8.0 (4.5, 12) preoperatively to 3.0 (1.0, 5.5), 4.0 (1.0, 6.5), and 5.0 (2.5, 9.0) at 1, 3, and 6 months postoperatively (Figure 5(a)). All postoperative HADS‐A scores were significantly lower than preoperative values (*p* < 0.05). The median HADS‐D scores decreased from 7.0 (4.5, 11.5) preoperatively to 2.0 (0.5, 5.0), 3.0 (1.0, 6.0), and 3.0 (1.5, 7.0) at 1, 3, and 6 months postoperatively (Figure 5(b)). All postoperative HADS‐D scores were significantly lower than preoperative values (*p* < 0.05).

Figure 5Postoperative HADS‐A scores (a) and HADS‐D scores (b) were significantly lower than preoperative levels. ^∗^ *p* < 0.05, ^∗∗^ *p* < 0.001, and ^∗∗∗^ *p* < 0.0001, compared to pre‐op.

**Figure:**
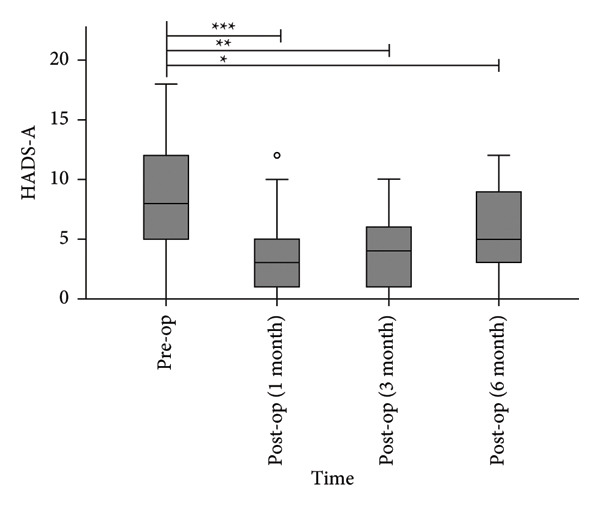
(a)

**Figure:**
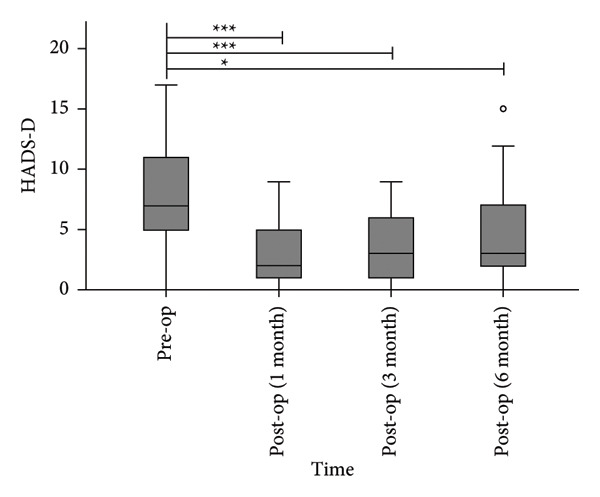
(b)

#### 3.1.4. PCS

**Figure 6:**
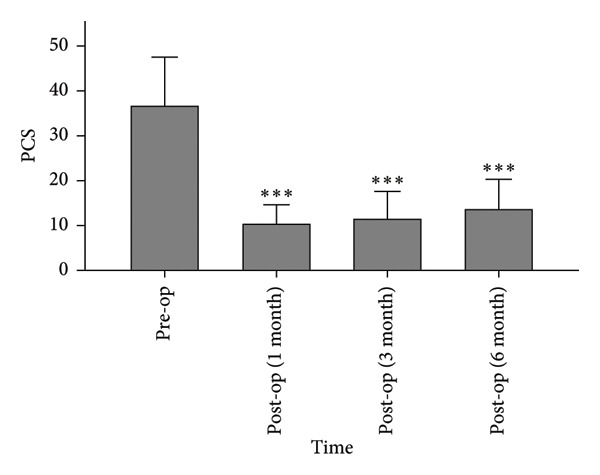
Postoperative PCS scores were significantly lower than preoperative levels. ^∗∗∗^ *p* < 0.0001, compared to pre‐op.

The PCS scores decreased from 36.58 ± 10.93 preoperatively to 10.22 ± 4.36, 11.46 ± 6.14, and 13.46 ± 6.80 at 1, 3, and 6 months postoperatively (Figure 6). Postoperative scores were significantly lower than preoperative values (*p* < 0.0001).

#### 3.1.5. Correlation Analysis of Symptom Improvement

ΔVAS, ΔICPI, and ΔICSI at 6 months postoperatively showed significant positive correlations with preoperative pudendal nerve diagnostic block outcomes, indicating that patients with better preoperative diagnostic block responses exhibited greater symptom improvement. No correlations were observed between ΔVAS, ΔICPI, and ΔICSI and HADS, PCS, or PSQ scores (Table 2).

**Table 2:** Correlation analysis of △VAS, △ICPI, and △ICSI with pudendal nerve blockade outcomes and related scales.

	△VAS		△ICPI		△ICSI	
	Correlation coefficient	*p* value	Correlation coefficient	*p* value	Correlation coefficient	*p* value
Pudendal nerve diagnostic block outcomes	0.610	0.000^∗^	0.712	0.000^∗^	0.681	0.000^∗^
HADS‐A (pre‐op)	0.062	0.735	0.168	0.294	0.226	0.156
HADS‐D (pre‐op)	0.055	0.764	0.121	0.452	0.185	0.247
PCS (pre‐op)	0.015	0.935	0.010	0.950	−0.065	0.688
PSQ (pre‐op)	0.330	0.065	0.287	0.069	0.304	0.053

^∗^Indicates statistical significance after Bonferroni correction (*p* < 0.05/5 = 0.01).

#### 3.1.6. Side Effects

Nine patients experienced buttock puncture site pain postoperatively, which resolved within 2 weeks. Two patients developed acute urinary retention within 2 hours postoperatively, which normalized after catheterization. No severe adverse events, such as infection, peripheral nerve injury, or urinary/defecation dysfunction, occurred.

## 4. Discussion and Conclusions

Patients with IC/BPS experience persistent physical and psychological distress. Perineal pain, in particular, severely compromises quality of life by limiting prolonged sitting or standing. Effective relief of perineal pain remains a critical unmet need. This retrospective study analyzed 51 female IC/BPS patients to evaluate the efficacy and safety of pudendal nerve PRF for perineal pain management. Results demonstrated significant reductions in pain scores following PRF. Concurrent declines in ICPI and ICSI scores suggest that PRF may also improve lower urinary tract symptoms, such as urinary frequency and urgency.

The mechanism underlying perineal pain in IC/BPS remains unclear but may involve organ cross‐sensitization between the bladder and pudendal nerve. The parasympathetic nerves innervating the detrusor muscle originate from the pelvic splanchnic nerves (S2‐S4), which share spinal segments with the pudendal nerve that provides sensory and somatic innervation to the urethra, vagina, vulva, and anal region. Convergent neural pathways at these spinal levels may facilitate cross‐talk. Animal studies have demonstrated bidirectional cross‐sensitization between the bladder and adjacent pelvic organs [10]. In IC/BPS, neurogenic bladder inflammation and urothelial dysfunction induce afferent hyperexcitability [11]. Persistent bladder afferent signaling may drive central sensitization, subsequently increasing pudendal nerve excitability and triggering perineal pain. The central mechanisms, however, require further experimental validation.

Neuromodulation has emerged as an established therapeutic approach for IC/BPS, primarily including sacral neuromodulation (SNM), pudendal neuromodulation, and tibial nerve stimulation. SNM remains the most frequently reported modality. Preclinical studies demonstrate that SNM reduces voiding frequency and suppresses nonvoiding bladder contractions in IC/BPS animal models [12]. Clinically, SNM has demonstrated significant improvements in pelvic pain, urinary frequency, and urgency in refractory IC/BPS patients [13]. However, limitations such as high costs, reoperation rates, late‐term failures, and adverse events necessitate more cost‐effective alternatives. In a comparative study, 79% of participants reported superior efficacy of pudendal neuromodulation over SNM for voiding dysfunction [14]. Another study found that 93.2% of IC/BPS patients with prior SNM failure responded to pudendal neuromodulation, with improvements in pelvic pain, frequency, and urgency [5]. These findings suggest that pudendal nerve–targeted neuromodulation may represent a more promising therapeutic strategy.

PRF, recognized for its minimally invasive nature, safety, efficacy, and cost‐efficiency, is widely utilized in managing neuropathic pain. Multiple studies have reported the effectiveness of pudendal nerve PRF in treating pudendal neuralgia [15–17]. This study similarly observed significant reductions in perineal pain among IC/BPS patients following pudendal nerve PRF, alongside improvements in lower urinary tract symptoms such as urinary frequency and urgency. The mechanisms underlying PRF‐mediated alleviation of perineal pain and urinary symptoms in IC/BPS remain unclear. Our previous work demonstrated that PRF applied to peripheral nerve trunks modulates ion channel expression in dorsal root ganglion neurons [18]. We hypothesize potential mechanisms as follows: (1) neuroplastic changes at the dorsal root ganglion and spinal levels reducing neuronal excitability, (2) central downregulation of bladder afferent hyperexcitability, and (3) normalization of urethral sphincter tone with subsequent inhibition of urethral mucosal nerve terminal excitation.

ΔVAS, ΔICPI, and ΔICSI at 6 months postoperatively demonstrated significant positive correlations with preoperative pudendal nerve diagnostic block efficacy. Pudendal nerve excitability may positively correlate with IC/BPS severity, as patients exhibiting superior diagnostic block responses demonstrated enhanced therapeutic responses to pudendal nerve PRF. These findings suggest pudendal nerve diagnostic block may serve as a predictive biomarker for PRF outcomes. No associations were observed between ΔVAS, ΔICPI, and ΔICSI and anxiety, depression, pain catastrophizing, or pain sensitivity, suggesting these parameters may reflect secondary manifestations rather than the underlying pathophysiology of IC/BPS.

Limitations of this study include the following: (1) Limited sample size and absence of a control group. Future randomized controlled trials with larger cohorts are required to comprehensively evaluate treatment efficacy and safety while ensuring generalizability. (2) The potential influence of Hunner lesion (HL) status on treatment outcomes was not assessed. As the HL and non‐HL subtypes of IC/BPS exhibit distinct pathological mechanisms and therapeutic responses, such an analysis is critical. However, in the present cohort, the number of patients with the HL subtype (*n* = 8) was substantially lower than that of the non‐HL subtype (*n* = 43). This significant disparity in sample size precluded a reliable subgroup analysis, as it would have been underpowered and prone to statistical bias. Future studies with a larger and balanced enrollment of HL patients are warranted to definitively determine the effect of HLs on the treatment outcomes of pudendal nerve PRF. (3) The findings of this study may not reflect the efficacy of pudendal nerve PRF in male patients with IC/BPS. Notably, while IC/BPS is more prevalent and often presents with greater pain intensity and extent in women [19], the condition in men represents a distinct clinical challenge. Male symptoms frequently overlap with chronic prostatitis/chronic pelvic pain syndrome (CP/CPPS), leading to frequent delays in diagnosis and potentially more complex management [20]. Therefore, future studies will include male participants to investigate the influence of gender on treatment outcomes, thereby providing valuable insights for adapting this therapeutic approach for men. (4) The 6‐month follow‐up period is insufficient for evaluating the long‐term efficacy of pudendal nerve PRF. Future investigations will incorporate extended follow‐up durations to thoroughly observe the enduring effects of the procedure.

In conclusion, pudendal nerve PRF demonstrates significant clinical efficacy and safety in alleviating perineal pain in patients with IC/BPS. Preoperative pudendal nerve diagnostic block outcomes may serve as a predictive biomarker for PRF therapeutic response.

## Ethics Statement

This study received ethical approval from the Institutional Review Board of Peking University People’s Hospital (Approval no. 2025PHB042‐001).

## Consent

No patient consent statement was necessary for this study.

## Disclosure

All authors approved the final manuscript.

## Conflicts of Interest

The authors declare no conflicts of interest.

## Author Contributions

Yiming Liu and Yi Feng designed and conducted the study, including patient recruitment and data analysis. Yiming Liu drafted the manuscript. Ou Wu, Yifan Yang, and Shunan Xie contributed to data collection.

## Funding

No funding was received to assist with the preparation of this manuscript.

## Data Availability

The data that support the findings of this study are available on request from the corresponding author.
